# Targeted DNA demethylation of the *Fgf21* promoter by CRISPR/dCas9-mediated epigenome editing

**DOI:** 10.1038/s41598-020-62035-6

**Published:** 2020-03-20

**Authors:** Nozomi Hanzawa, Koshi Hashimoto, Xunmei Yuan, Kenichi Kawahori, Kazutaka Tsujimoto, Miho Hamaguchi, Toshiya Tanaka, Yuya Nagaoka, Hiroshi Nishina, Sumiyo Morita, Izuho Hatada, Tetsuya Yamada, Yoshihiro Ogawa

**Affiliations:** 1 Department of Molecular Endocrinology and Metabolism, Graduate School of Medical and Dental Sciences, Tokyo Medical and Dental University, 1-5-45 Yushima, Bunkyo-ku, Tokyo 113-8510 Japan; 2 Department of Preemptive Medicine and Metabolism, Graduate School of Medical and Dental Sciences, Tokyo Medical and Dental University, 1-5-45 Yushima, Bunkyo-ku, Tokyo 113-8510 Japan; 3 Department of Diabetes, Endocrinology and Hematology, Dokkyo Medical University Saitama Medical Center, 2-1-50 Minami-Koshigaya, Koshigaya, Saitama 343-8555 Japan; 4 Laboratories for Systems Biology and Medicine (LSBM), Research Center for Advanced Science and Technology (RCAST), The University of Tokyo, Tokyo, 153-8904 Japan; 5 Department of Developmental and Regenerative Biology, Medical Research Institute, Tokyo Medical and Dental University, 1-5-45 Yushima, Bunkyo-ku, Tokyo 113-8510 Japan; 6 Laboratory of Genome Science, Biosignal Genome Resource Center, Institute for Molecular and Cellular Regulation, Gunma University, 3-39-15 Showa-machi, Maebashi, Gunma 371-8512 Japan; 7 Department of Molecular and Cellular Metabolism, Graduate School of Medical and Dental Sciences, Tokyo Medical and Dental University, 1-5-45 Yushima, Bunkyo-ku, Tokyo 113-8510 Japan; 8 Department of Medicine and Bioregulatory Science, Graduate School of Medical Sciences, Kyushu University, 3-1-1 Maidashi, Higashi-ku, Fukuoka 812-8582 Japan

**Keywords:** DNA methylation, Obesity

## Abstract

Recently, we reported PPARα-dependent DNA demethylation of the *Fgf21* promoter in the postnatal mouse liver, where reduced DNA methylation is associated with enhanced gene expression after PPARα activation. However, there is no direct evidence for the effect of site-specific DNA methylation on gene expression. We employed the dCas9-SunTag and single-chain variable fragment (scFv)-TET1 catalytic domain (TET1CD) system to induce targeted DNA methylation of the *Fgf21* promoter both *in vitro* and *in vivo*. We succeeded in targeted DNA demethylation of the *Fgf 21* promoter both in Hepa1-6 cells and PPARα-deficient mice, with increased gene expression response to PPARα synthetic ligand administration and fasting, respectively. This study provides direct evidence that the DNA methylation status of a particular gene may determine the magnitude of the gene expression response to activation cues.

## Introduction

In mammalian cells, DNA methylation is a major epigenetic modification, which regulates gene expression without alteration of the DNA sequence and thus plays a pivotal role in a myriad of physiological and pathological processes, including cell development and differentiation, genome imprinting, and tumorigenesis^1^. We reported previously that the DNA methylation status of hepatic metabolic genes dynamically changes in early life, especially during the suckling period, thereby sequentially developing metabolic function in the liver to adapt to the drastic changes in the major nutrition source^2–4^.

Peroxisome proliferator-activated receptor-α (PPARα) is a nuclear receptor and a key regulator of hepatic lipid metabolism, which is activated by milk lipids as ligands at the onset of lactation. PPARα governs the transcription of major hepatic metabolism-related genes, and the activation of PPARα physiologically leads to DNA demethylation of fatty acid β-oxidation genes in the postnatal mouse liver^3,4^. Of note, administration of a synthetic PPARα ligand to mouse dams during the perinatal period induced enhanced reduction of DNA methylation of PPARα target genes in the offspring liver, suggesting that the DNA methylation status of PPARα target genes can be modulated with ease during the perinatal period^3,4^. A genome-wide analysis of DNA methylation revealed that a few PPARα target genes undergo ligand-activated, PPARα-dependent DNA demethylation during the perinatal period, and the DNA hypomethylation status of these persists into adulthood. Among these genes, which may be referred to as “epigenetic memory genes,” we focused on fibroblast growth factor 21 (FGF21), which is a metabolic hormone derived from the liver and a master regulator of glucose and lipid metabolism^5–7^. We found that the DNA methylation status of the mouse FGF21 gene (*Fgf21*) promoter, which is established during the suckling period, is maintained into adulthood^4^. Notably, reduced DNA methylation correlated with enhanced induction of hepatic *Fgf21* expression in response to PPARα activation cues, which may partly explain the attenuation of obesity induced by high-fat diet administration in adulthood^4^. However, because other PPARα target genes also undergo PPARα-dependent DNA demethylation, direct causative effects of DNA methylation status of the *Fgf21* promoter *per se* for the metabolic phenotypes mentioned above are unclear. Moreover, nonspecific DNA methyltransferase inhibitors such as 5-aza-2′-deoxycytidine demethylate the genome globally^8^; therefore, it is hard to induce DNA demethylation of a specific gene.

Clustered regularly interspaced short palindromic repeats/CRISPR-associated protein-9 nuclease (CRISPR/Cas9) is a unique technology for genome editing^9^. Single guide RNA (sgRNA)-mediated DNA targeting, followed by Cas9 endonuclease-mediated DNA cleavage enables site-specific genome editing^10^.

A deactivated or dead Cas9, known as dCas9 possesses mutations in the catalytic domain of Cas9, whose endonuclease activity is removed but can bind a specific site in the genome defined by an sgRNA. The CRISPR/dCas9 system can be employed as a DNA-binding platform for generating chimeric versions of dCas9 fused with epigenetic modifiers, which may achieve targeted epigenome editing^11–13^.

Recently, a new technique to edit the DNA methylation status of specific genes using dCas9 fused with a SunTag has been developed^14,15^. SunTag consists of a tandem repeat of five copies of the 19 amino-acid GCN4 peptides separated by 22 amino-acid linkers, which can recruit several copies of the single-chain variable fragment (scFv) of the anti-GCN4 antibody-fused Ten Eleven Translocation 1 catalytic domain (TET1CD) fusion protein^15,16^, thereby promoting DNA demethylation^15^. In this study, we employed the dCas9-SunTag and scFv-TET1CD epigenome editing system to induce targeted DNA demethylation of the *Fgf21* promoter, thereby exploring the physiological and functional significance of *Fgf21* DNA methylation status.

## Results

### Transient transfection in Hepa1-6 cells

**Figure 1:**
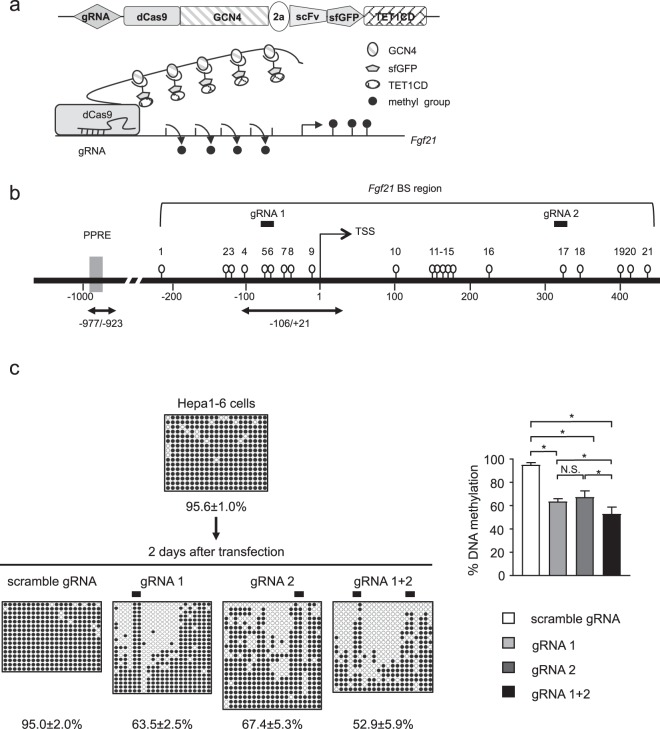
Targeted DNA demethylation of the *Fgf21* promoter using the dCas9-SunTag and scFv-TET1CD system in Hepa1-6 cells. (**a**) Schematic figure of the dCas9-SunTag and scFv-TET1CD system. (**b**) Schematic representation of the promoter region of *Fgf21*. Open circles and gray boxes indicate CpG sites and PPAR response elements (PPREs), respectively. The region analyzed using bisulfite sequencing (BS) encompassing the transcription start site (TSS) is indicated. Bidirectional arrows indicate primers used for ChIP-qPCR analysis (Fig. 5c). Solid bars indicate selected target gRNAs (gRNA1 and gRNA2). (**c**) BS analysis of DNA methylation status of the *Fgf21* promoter after transient transfection of the system with gRNAs in Hepa1-6 cells. Closed and open circles indicate methylated and unmethylated CpGs, respectively. Representative data of four independent experiments are shown. Statistical analyses were performed using the Mann–Whitney *U*-test. Data are expressed as mean ± SD. *P < 0.05; N.S., not significant between the denoted pairs.

To achieve targeted DNA demethylation of the *Fgf21* promoter, we introduced the dCas9-SunTag and scFv-TET1CD epigenome editing system (Fig. 1a) into cultured cells by transient transfection.

*In silico* searches identified 21 CpG sites around the transcription start site (TSS) of *Fgf21* (Fig. 1b)^4,17^. We sought cell lines in which the *Fgf21* promoter was highly DNA methylated. We performed bisulfite sequencing (BS) analysis of the *Fgf21* promoter using several cell lines and found that the *Fgf21* promoter was most highly DNA methylated in mouse hepatocyte-derived Hepa1-6 cells (Fig. 1c). Therefore, in this study, we employed Hepa1-6 cells for targeted DNA demethylation of the *Fgf21* promoter using the dCas9-SunTag and scFv-TET1CD system. We selected gRNAs in the upstream and downstream regions of the TSS as gRNA1 and gRNA2, which bound to CpG sites #5–6 and #17, respectively (Fig. 1b). A scramble gRNA was used as a control.

In this study, 2 days after transfection, we performed cell sorting using FACS (Supplementary Fig. 1). The transfection efficiency was approximately 17%, as shown by GFP-positive cells (Supplementary Fig. 1). We determined the extent of targeted DNA demethylation in the promoter region of *Fgf21* using BS analysis. Epigenome editing with gRNA1 significantly induced DNA demethylation in the *Fgf21* promoter region relative to the scramble gRNA (Fig. 1c). It was noteworthy that DNA demethylation was spared at CpG sites #5 and #6, where gRNA1 bound (Fig. 1c). Epigenome editing with gRNA2 induced DNA demethylation in the *Fgf21* promoter region to the same extent as gRNA1, but with a different DNA demethylation pattern (Fig. 1c). We also co-transfected gRNA1 and gRNA2 (gRNA1+2), resulting in additively increased levels of DNA demethylation (Fig. 1c).

To confirm if the overexpression of TET1CD alone induced non-specific global genome-wide DNA demethylation, known as an ‘off-target’ effect, we analyzed the DNA methylation status of potential regions that have the similarity to gRNA1 and gRNA2 sequences, revealing that DNA methylation was induced at CpG sites only in one region among these regions (Supplementary Fig. 2), which suggested that the ‘off-target’ effects from the dCas9-SunTag and scFv-TET1CD system appeared to be minimal.

### Time course of transient transfection-induced DNA demethylation

**Figure 2:**
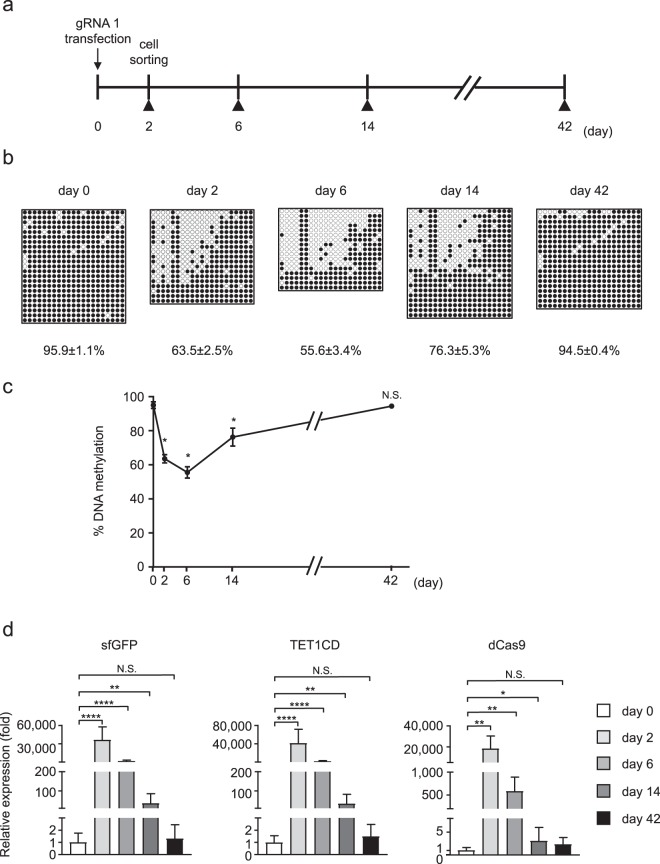
Time course analysis of targeted DNA demethylation of the *Fgf21* promoter induced by the dCas9-SunTag and scFv-TET1CD system via transient transfection in Hepa1-6 cells. (**a**) Experimental protocol for the transient transfection of the system with gRNA1. Closed triangles indicate BS analysis. (**b**) BS analysis of the *Fgf21* promoter at the designated time points. Representative data of four independent experiments are shown. %DNA methylation is indicated below as mean ± SD. (**c**) Graphic presentation of statistical analysis of the BS data (*n* = 4 at each time point). Data are expressed as mean ± SD. Statistical analyses were performed using the Mann–Whitney *U*-test. *P < 0.05; N.S., not significant vs. day 0. (**d**) Gene expression levels of sfGFP, TET1CD, and dCas9 at the designated time points. Statistical analyses were performed using the Mann–Whitney *U*-test. Data are expressed as mean ± SD. n = 6–9 per group. *P < 0.05; **P < 0.01; ****P < 0.0001; N.S., not significant vs. day 0.

Next, we examined the time course of DNA demethylation of the promoter region of *Fgf21* through use of the dCas9-SunTag and scFv-TET1CD system. Following the experimental protocol (Fig. 2a), the DNA methylation status, once induced, was retained for 6 days after transfection, after which we passaged the transfected cells every 3 days (Fig. 2a). At day 14, after the second passage, we found moderate DNA re-methylation of the promoter region of *Fgf21* (Fig. 2b). At day 42, there was marked DNA re-methylation, which did not differ significantly from that at day 0, indicating that transient transfection-induced DNA demethylation was not retained during passage (Fig. 2b,c). We also found that the gene expression levels of sfGFP, TET1CD and dCas9 in Hepa1-6 cells declined in a time-dependent manner (Fig. 2d). These observations, taken together, suggested that DNA re-methylation was due mostly to the absence of the transfected construct.

### Dnmt1 and Dnmt3a contribute to re-methylation in the *Fgf21* promoter region

**Figure 3:**
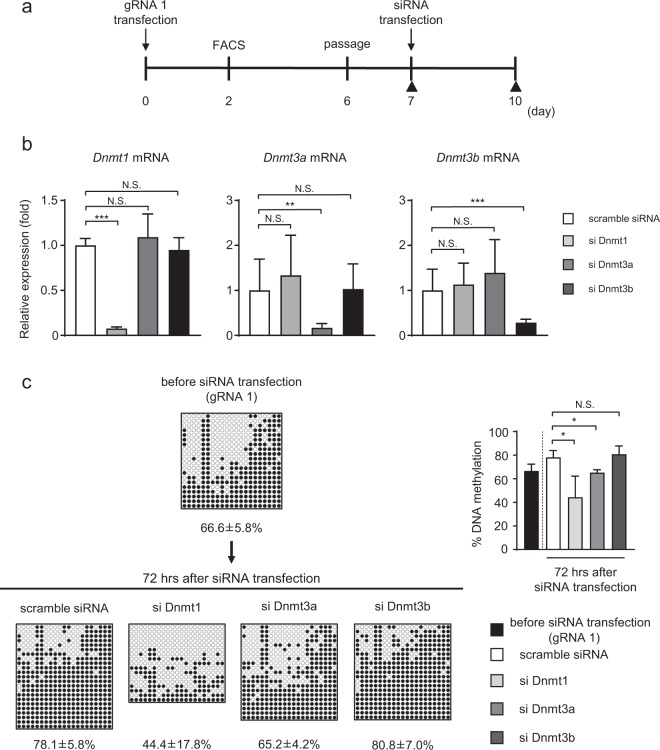
Transfection of Dnmts siRNA. (**a**) Experimental protocol of the transient transfection system with gRNA1 and Dnmts siRNA. Closed triangles indicate BS analysis. (**b**) Gene expression levels of Dnmts 72 h after transfection with scramble or Dnmts siRNA in Hepa1-6 cells. Statistical analyses were performed using the Mann–Whitney *U*-test. Data are expressed as mean ± SD. n = 7–9 per group. **P < 0.01; ***P < 0.001; N.S., not significant vs. scramble siRNA. (**c**) (Left panel) BS analysis of the *Fgf21* promoter at the designated time points. Representative data of four independent experiments are shown. %DNA methylation is indicated below as mean ± SD. (Right panel) Graphic presentation of statistical analysis of the BS data (*n* = 4 at each time point). Statistical analyses were performed using the Mann–Whitney *U*-test. Data are expressed as mean ± SD. *P < 0.05; N.S., not significant vs. scramble siRNA.

To elucidate the mechanism by which DNA methylation was re-induced in the *Fgf21* promoter region after transient transfection of the dCas9-SunTag and scFv-TET1CD system, we employed small interference (si) RNA of DNA methyltransferases (Dnmts) (Dnmts siRNA). We subcultured Hepa1-6 cells at day 6 after transient transfection with the dCas9-SunTag and scFv-TET1CD system in 10-cm dishes and transfected 120 pmol/dish of scramble or Dnmts siRNA into the cells at day 7 (Fig. 3a). At day 10, we extracted genomic DNA and RNA from the cells and performed qPCR and BS analysis (Fig. 3a). Confirming the siRNA effects on the repression of the gene expression of Dnmts (Fig. 3b), we found that the DNA methylation ratio was significantly lower in cells with Dnmt1 and Dnmt3a siRNA than in those transfected with scramble siRNA, suggesting that Dnmt1 and Dnmt3a contributed to DNA re-methylation (Fig. 3c).

### Stable transfection in Hepa1-6 cells

**Figure 4:**
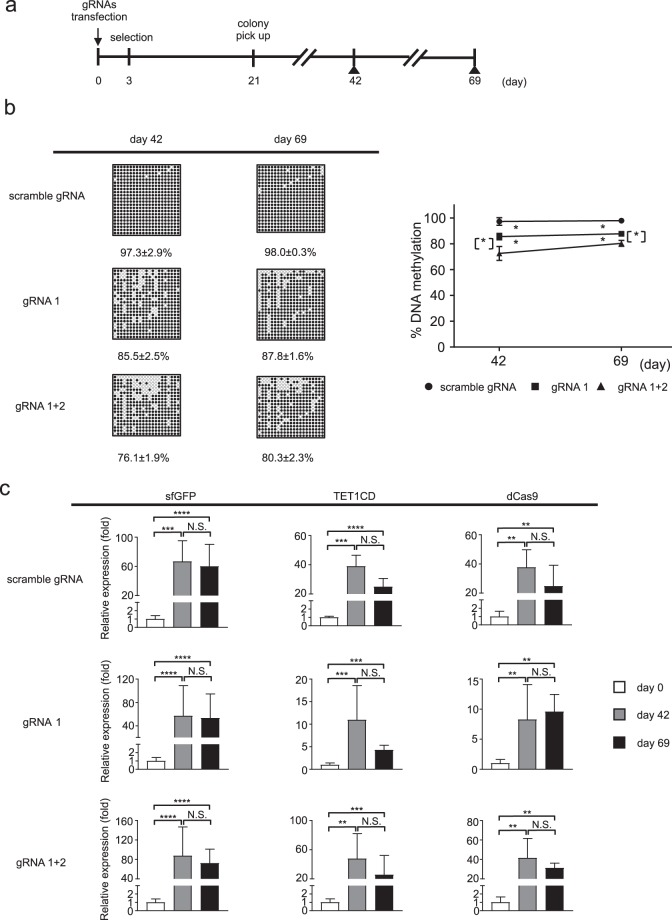
Time course analysis of targeted DNA demethylation of the *Fgf21* promoter induced using the dCas9-SunTag and scFv-TET1CD system via stable transfection in Hepa1-6 cells. (**a**) Experimental protocol of the stable transfection system with gRNAs (scramble gRNA, gRNA1, and gRNA1+2). Closed triangles indicate BS analysis. (**b**) BS analysis of the *Fgf21* promoter at the designated time points. Representative data of four independent experiments are shown (left panel). %DNA methylation is indicated below as mean ± SD. Graphic presentation of statistical analysis of the BS data. (*n* = 4 at each time point) (right panel). Statistical analyses were performed using the Mann–Whitney *U*-test. Data are expressed as mean ± SD. *P < 0.05 vs. scramble gRNA. [*] *P* < 0.05 between gRNA1 and gRNA1+2. (**c**) Gene expression levels of sfGFP, TET1CD, and dCas9 at the designated time points. Statistical analyses were performed using the Mann–Whitney *U*-test. Data are expressed as mean ± SD. n = 6–9 per group. **P < 0.01; ***P < 0.001; ****P < 0.0001; N.S., not significant between the denoted pairs.

Because targeted DNA demethylation of the *Fgf21* promoter induced by transient transfection was not maintained on a long-term basis, we established Hepa1-6 cell lines with stable expression of the system and examined whether targeted DNA demethylation of the *Fgf21* promoter can persist (Fig. 4a). Because we found no significant difference in DNA methylation rate with between gRNA1 and gRNA2 in the transient transfection experiments (Fig. 1c), we employed gRNA1 and gRNA1+2 to establish stable cell lines.

Stable transfection of the epigenome editing with gRNA1 and gRNA1+2 significantly induced DNA demethylation of the *Fgf21* promoter relative to that with scramble gRNA, and the extent of DNA demethylation was significantly enhanced with gRNA1+2 more than with gRNA1 (Fig. 4b). In this case, DNA methylation status persisted for several cell passages. Accordingly, the mRNA expression levels of sfGFP, TET1CD and dCas9 were maintained throughout the experimental protocol (Fig. 4c). We also evaluated the copy number after random integration of the dCas9 cassette at day 42 and found that no significant difference in the copy number among gRNAs (Supplementary Fig. 3).

### Impact of targeted DNA demethylation on the gene expression response *in vitro*

**Figure 5:**
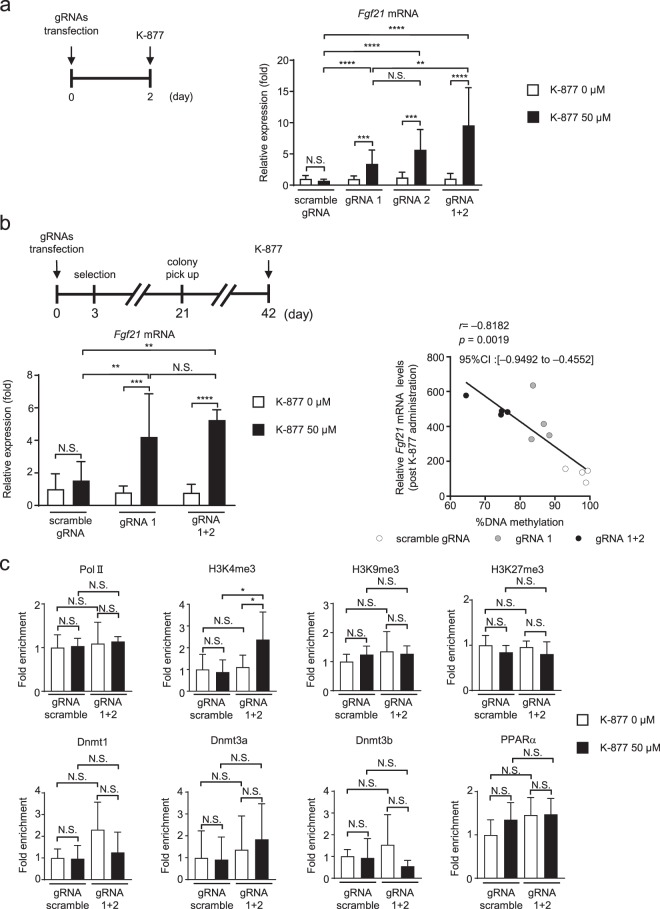
*Fgf21* mRNA expression in Hepa1-6 cells with targeted DNA demethylation of the *Fgf21* promoter induced using the dCas9-SunTag and scFv-TET1CD system. (**a**) Experimental protocol of the transient transfection system with gRNAs (scramble gRNA, gRNA1, gRNA2, and gRNA1+2) and administration of SPPARMα, K-877 (left panel). *Fgf21* mRNA expression levels after the administration of K-877 (right panel). n = 7–9 per group. (**b**) Experimental protocol of the stable transfection system with scramble gRNA, gRNA 1, and gRNA 1+2 and administration of K-877 (upper panel). *Fgf21* mRNA expression levels after the administration of K-877 (lower left panel). n = 6–7 per group. Correlation between *Fgf21* mRNA levels post K-877 administration and %DNA methylation (*n* = 12) (lower right panel). Statistical analyses were performed using Spearman’s rank correlation coefficient. CI: Confidence interval. (**c**) ChIP assays of Pol II, histone markers, Dnmts, and PPARα. Primers amplifying the regions −106 to +21 (for Pol II, histone markers, and Dnmts) and of −977 to −923 bp (for PPARα) were used for ChIP-qPCR analysis (Fig. 1b). n = 5–6 per group. Statistical analyses were performed using the Mann–Whitney *U*-test. Data are expressed as mean ± SD. *P < 0.05, **P < 0.01; ***P < 0.001; ****P < 0.0001; N.S., not significant between the denoted pairs.

Generally speaking, DNA demethylation is associated with increased mRNA expression. Therefore, we examined mRNA expression in Hepa1-6 cells after being transfected transiently with either targeted or non-targeted constructs (Fig. 5a). In this study, there was no significant difference in steady-state *Fgf21* mRNA levels between cells transfected with non-targeted scramble gRNA and those with targeted constructs (gRNA1, 2, and 1+2) (Fig. 5a). To further explore if targeted DNA demethylation of the *Fgf21* promoter affected mRNA expression responses, we examined the impact of K-877, a novel selective PPARα modulator (SPPARMα)^18^ on the *Fgf21* mRNA expression response. Upon K-877 administration, *Fgf21* mRNA expression was significantly increased, when transiently transfected with either gRNA1, gRNA2, or gRNA1+2 relative to scramble gRNA. Similar data were obtained using Hepa1-6 cell lines that stably expressed the dCas9-SunTag and scFv-TET1CD system with gRNA1 and gRNA1+2 (Fig. 5a,b).

Even though we found no significant difference in *Fgf21* mRNA levels after K-877 administration between gRNA1 and gRNA1+2, a correlation plot showed a negative correlation between the degree of DNA methylation (%DNA methylation) and the induction of gene expression (Fig. 5b).

These observations, taken together, suggested that the DNA methylation status of the *Fgf21* promoter may not determine the steady-state *Fgf21* mRNA expression level but instead the mRNA expression response to PPARα activation.

Using chromatin immunoprecipitation (ChIP) assays, we evaluated the enrichment of RNA polymerase II (Pol II), histone markers, Dnmts, and PPARα (Fig. 5c). H3K4me3, a transcriptionally active histone marker, was significantly enriched in the *Fgf21* promoter region in cells transfected with gRNA1+2 relative to scramble gRNA, upon transient PPARα activation with K-877. On the other hand, the levels of the repressive histone markers, H3K9me3 and H3K27me3 were approximately similar between those cells transfected with gRNA1+2 and scramble gRNA. Successful enrichment of histone markers was verified using control ChIP assays, targeting the promoter region of the GAPDH (for H3K4me3) and MyoD (for H3K9me3 and H3K27me3) genes (Supplementary Fig. 4). Enrichment of Pol II and Dnmts was also approximately similar in both groups. Moreover, upon PPARα activation, the recruitment of PPARα was similar in both groups.

### Targeted DNA demethylation of the *Fgf21* promoter in the adult mouse liver

**Figure 6:**
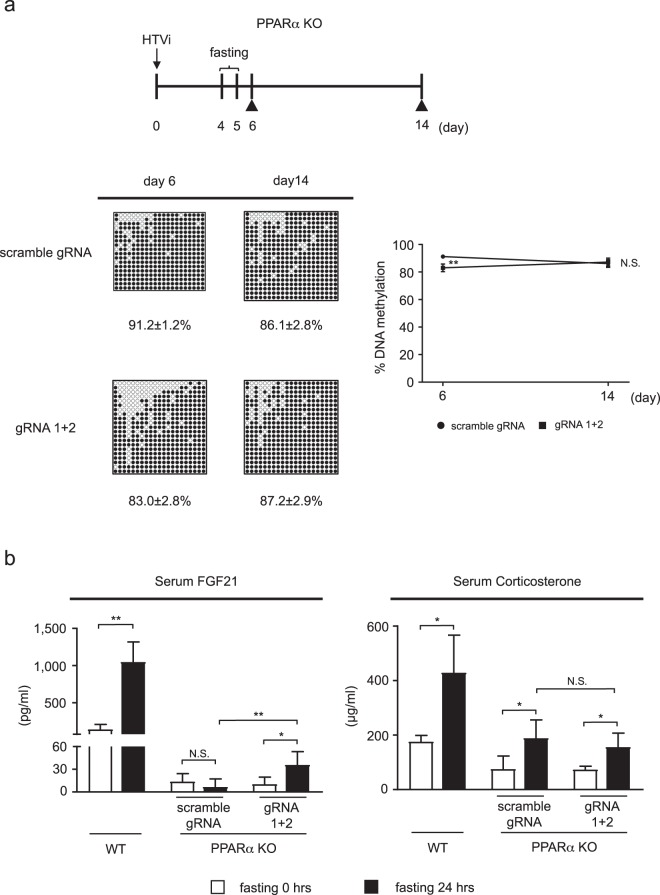
Targeted DNA demethylation of the *Fgf21* promoter induced by the dCas9-SunTag and scFv-TET1CD system *in vivo*. (**a**) Experimental protocol of the induction system with scramble gRNA and gRNA 1+2 in the liver of PPARα KO mice via HTVi (upper panel). Closed triangles indicate BS analysis. BS analysis of the *Fgf21* promoter at day 6 (n = 5–9 per group) and day 14 (n = 6–7 per group). Representative data of each time point are shown (lower left panel). %DNA methylation is indicated below as mean ± SD. Graphic presentation of statistical analysis of the BS data (lower right panel). Statistical analyses were performed using the Mann–Whitney *U*-test. Data are expressed as mean ± SD. *P < 0.05; N.S., not significant vs. scramble gRNA. (**b**) Serum FGF21 (left panel, n = 4–6 per group) and corticosterone (right panel, n = 4–7 per group) concentrations after 24-h fasting. Statistical analyses were performed using the Mann–Whitney *U*-test. Data are expressed as mean ± SD. *P < 0.05; **P < 0.01; N.S., not significant between the denoted pairs.

To examine if targeted DNA demethylation of the *Fgf21* promoter occurs *in vivo*, we introduced the dCas9-SunTag and scFv-TET1CD system into the liver of wild-type (WT) mice using the hydrodynamic tail vein injection (HTVi) method (Supplementary Fig. 5a). In this study, HTVi achieved exogenous gene expression in about 4% of hepatocytes (Supplementary Fig. 5b). However, BS analysis (Supplementary Fig. 6a) revealed no significant difference in DNA methylation of the *Fgf21* promoter between gRNA1+2 and scramble gRNA (Supplementary Fig. 6b). This was partly because DNA demethylation of the *Fgf21* promoter occurred sufficiently in the adult WT mouse liver, where induction of the epigenome editing system by HTVi did not exhibit significantly increased DNA demethylation. Because DNA demethylation of the *Fgf21* promoter occurred via a PPARα-dependent mechanism, we next employed PPARα-deficient (KO) mice. At day 6 after HTVi (Fig. 6a), BS analysis revealed increased epigenome editing with gRNA1+2 in the *Fgf21* promoter region relative to scramble gRNA (Fig. 6a). At day 14 after HTVi, we performed BS analysis and found re-methylation of DNA in the *Fgf21* promoter region, with no significant difference between gRNA1+2 and scramble gRNA (Fig. 6a).

### Impact of targeted DNA demethylation on the gene expression response *in vivo*

Next, we examined if targeted DNA demethylation of the *Fgf21* promoter affected gene expression in the liver *in vivo*. There is evidence that fasting-induced *Fgf21* expression involves a PPARα-independent mechanism^17,19^, which may be mediated at least in part by adrenal corticosterone^20^. Because we employed PPARα-KO mice, we examined the effect of fasting on the *Fgf21* expression response. At day 4 after HTVi, mice were used for analysis after 24-h fasting (Fig. 6a). In this study, serum FGF21 concentrations were increased in WT mice after 24-h fasting (Fig. 6b). In response to 24-h fasting, serum FGF21 concentrations were slightly, but significantly, increased in PPARα-KO mice injected with gRNA1+2. On the other hand, serum FGF21 concentrations were unchanged in PPARα-KO mice injected with scramble gRNA. Moreover, serum FGF21 concentrations in response to 24-h fasting in PPARα-KO mice injected with gRNA1+2 were significantly higher than those injected with scramble gRNA (Fig. 6b). In this study, serum corticosterone concentrations were increased in both PPARα-KO mice injected with gRNA1+2 and those injected with scramble gRNA in response to 24-h fasting, with no significant difference between the groups (Fig. 6b). These observations suggested that fasting-induced serum FGF21 concentrations are due to increased responsiveness rather than increased serum corticosterone concentrations in PPARα-KO mice with targeted DNA demethylation of the *Fgf21* promoter.

## Discussion

In this study, we successfully induced targeted DNA demethylation of the *Fgf21* promoter both *in vitro* and *in vivo* using the dCas9-SunTag and scFv-TET1CD system. Even though we concluded that off-target effects might be minimal based on our data, this should be proven using a whole-genome analysis, which was not performed in the current study, and this was a limitation in the analysis of off-target effects.

Notably, the degree of DNA demethylation of the *Fgf21* promoter varied depending upon the combination of gRNAs used. In the current study, we employed gRNA1 for targeting the upstream region of the TSS, which is the promoter region, and gRNA2 for the downstream region, which is the gene body. Even though the physiological function of DNA methylation in the gene body has not yet been fully elucidated, it has been shown that DNA methylation is a marker for some actively transcribed genes^21^ and that DNA demethylation of the gene body induced by 5-aza-2′-deoxycytidine treatment caused reduced gene expression in some cancer-related genes^22^. However, DNA demethylation induced by epigenome editing with gRNA2 showed an enhanced gene expression response upon K-877 administration (Fig. 5a). Moreover, in our previous report^4^, we found that enhanced DNA demethylation via ligand-activated PPARα specifically occurs at CpG sites in the gene body of *Fgf21* in the perinatal mouse liver, and DNA demethylation of these CpG sites was correlated with the induction of gene expression. Taken together, these data suggest that DNA methylation of the gene body of *Fgf21* exerts repressive effects on gene expression.

This study also examined a detailed time course analysis of targeted DNA demethylation of a particular gene *in vitro* with the DNA methylation pattern examined using BS analysis, thereby providing a unique experimental opportunity with which to examine the molecular basis for epigenome editing using the dCas9-SunTag and scFv-TET1CD system.

We demonstrated previously that the DNA methylation status of the *Fgf21* promoter, which is established in the postnatal mouse liver during the suckling period, is maintained into adulthood^4^. However, in this study, targeted DNA demethylation of the *Fgf21* promoter by transient transfection was not maintained, but that by stable transfection was maintained in Hepa1-6 cells even over several passages. Because mRNA expression of sfGFP, TET1CD and dCas9 decreased in a time-dependent manner after transient transfection, we speculated that DNA re-methylation found in the transient transfection experiments might be due to the absence of the TET1-CD constructs after several passages.

Previous studies using CRISPR-based epigenome editing also reported the effects of targeted DNA demethylation using transient transfection. Wang *et al*. reported that targeted DNA demethylation could last up to 23 days^23,24^, whereas Marx *et al*. reported that increased gene expression by targeted DNA demethylation was maintained up to 80 days^23,24^. Furthermore, Josipović *et al*. employed a different TET1-dCas9 construct from ours and induced targeted DNA demethylation. They also performed a time course study after transient transfection and found that even though almost no construct was present on 8th day and the dCas9 protein was undetectable by the 11th day after transfection, the effect of DNA demethylation persisted until day 30 after transfection. Taken together with our data, these data suggest that the effect of targeted DNA demethylation by transient transfection may depend on the target genes and the TET1-dCas9 system^25^. However, DNA re-methylation over these time periods has never been reported. In the current study, we provided the first evidence that Dnmt1 and Dnmt3a play a pivotal role in DNA re-methylation after the induction of DNA demethylation using the dCas9-SunTag and scFv-TET1CD system. These data suggested that upon artificial DNA demethylation, Dnmt1 and Dnmt3a can re-induce DNA methylation to maintain homeostatic DNA methylation status.

It is known that TET proteins compete with Dnmts in the recruitment to gene promoters and interfere with DNA methylation by Dnmts^26,27^. Therefore, despite artificial targeted DNA demethylation using TET1CD in the current study, it is conceivable that Dnmts induce DNA re-methylation of the *Fgf21* promoter in the absence of TET1CD constructs from Hepa1-6 cells.

We found that DNA demethylation was significantly enhanced with gRNA1+2 than with gRNA1 or gRNA2 in both transient and stable transfection of the system, which may not be attributed to the amount of the transfected construct. It has been known that the combination of several gRNAs, which target different sites would be more effective in CRISPR/dCas9-based genome editing than single gRNA than single gRNA in previous reports^16,28^. Since gRNA1 and gRNA2 target different sites in *Fgf21* promoter, the combination of these gRNAs, which is gRNA1+2 may have shown the enhanced DNA demethylation.

In contrast to the rapidly growing number of targeted epigenome editing studies in the native chromatin context, only a few studies have reported successful editing of the epigenome in adult animals^13,16,23,29^. In this regard, it is of note that we succeeded in inducing targeted DNA demethylation of the *Fgf21* promoter using the dCas9-SunTag and scFv-TET1CD system in PPARα-KO mouse livers using HTVi. It has been reported that the transfection efficiency of HTVi in mouse liver is 5–40%^30–32^, indicating that the transfection efficiency (about 4%) with pPlatTET-gRNA2 all-in-one vector was less than expected. However, the transfection efficiency of HTVi in mouse liver mainly depended on the size of the injected construct. Indeed, when we performed HTVi in WT mice using pMAX-GFP (Lonza, Basel, Switzerland), which is about 3.5 kb in size, we obtained about 16% GFP-positive cells (Supplementary Fig. 5b), suggesting that HTVi was performed successfully. On the other hand, the all-in-one vector, which is about 14 kb in size, is much larger than pMAX-GFP. Therefore, we speculated that the low transfection efficiency with the all-in-one vector might be due to its size despite successful injection. Nonetheless, we achieved significant DNA demethylation of the *Fgf21* promoter in PPARα-KO mice, suggesting that even the 4% transfection efficiency might be effective for DNA demethylation induced by HTVi, which may be a method worth considering to induce targeted DNA demethylation of particular genes in the liver.

On the other hand, the DNA methylation status of the *Fgf21* promoter in the liver induced using HTVi was not maintained over the long term. Presumably, because HTVi is a transient transfection method, the absence of the construct may occur, and as we found *in vitro*, Dnmt1 and Dnmt3a may trigger DNA re-methylation. Physiologically, TET expression levels in the mouse liver are high, with a peak during the suckling period and decreasing thereafter^4^, suggesting that an unknown mechanism other than TET enzyme activity may work to maintain DNA demethylation of the *Fgf21* promoter *in vivo*. Thus, the differences between physiological and artificial *Fgf21* promoter DNA demethylation are yet to be clarified, which is a limitation of the current study.

A couple of previous studies reported that targeted DNA demethylation significantly increased steady-state gene expression^23,24,29^. In this study, targeted DNA demethylation of the *Fgf21* promoter induced a gene expression response to PPARα activation rather than steady-state gene expression in Hepa1-6 cells. Similar data were obtained using PPARα-KO mice *in vivo*. Considering that the *Fgf21* promoter is highly methylated both in Hepa1-6 cells and PPARα-KO mice, thereby resulting in low gene expression levels both *in vitro* and *in vivo*, altered epigenetic regulation of *Fgf21* expression via targeted DNA demethylation may only be detected by activation cues such as pharmacologic activation of PPARα and fasting. In our previous report^4^, we found that about 10% change in DNA methylation status of the *Fgf* 21 promoter, which was a similar extent to obtained in the current study, resulted in about 3-fold change in gene expression in response to fasting, suggesting that subtle but significant differences in DNA methylation may induce substantial differences in gene expression.

Epigenetic modulation as a result of DNA methylation often serves as an on-off switch for gene expression^33^. On the other hand, the DNA methylation status of the *Fgf21* promoter may serve as a determinant of the magnitude of the gene expression response to activation cues, which was suggested in our previous study^4^ and was supported by targeted DNA demethylation of the *Fgf21* promoter in the current study.

In this regard, active histone markers were significantly enriched in the *Fgf21* promoter region upon activation cues in Hepa1-6 cells, which was consistent with our previous data *in vivo*^4^. This also suggested that the induction of gene-specific DNA demethylation resulted in dynamic alterations in the patterns of H3K4me3^34^.

Unfortunately, we did not examine the metabolic phenotypes of mice with targeted DNA demethylation of the *Fgf21* promoter; this was partly because we only examined the impact of targeted DNA demethylation of the *Fgf21* promoter *in vivo* and partly because DNA methylation status, once induced, was not maintained on a long-term basis. In this regard, dCas9-SunTag and scFv-TET1CD transgenic mice, which may be referred to as “a gene-specific epigenetically modified animal”, are useful to address this issue. The phenotypic impact of targeted DNA demethylation of the *Fgf21* promoter *in vivo* must await further investigation.

In conclusion, this study provides the first evidence that targeted DNA methylation of the *Fgf21* promoter can be achieved using the dCas9-SunTag and scFv-TET1CD system *in vitro* and *in vivo*, which can be easily applied to examine the causality of disease-associated DNA methylation events and evaluate the consequences after targeted reversal of the DNA methylation status, possessing great potential for future research into novel therapies. Moreover, the data from this study suggest that the DNA methylation status of a particular gene may determine the magnitude of the gene expression response to activation cues, which can be a novel paradigm of DNA methylation.

## Materials and methods

### Animals

All animal experiments were approved by the Tokyo Medical and Dental University Committee on Animal Research (No. A2018-197A). All methods involving animals were performed in accordance with the relevant guidelines and regulations. All mice were treated in accordance with the Fundamental Guidelines for Proper Conduct of Animal Experiment and Related Activities in Academic Research Institutions under the jurisdiction of the Ministry of Education, Culture, Sports, Science and Technology of Japan. All efforts were made to minimize suffering and to reduce the number of animals used.

Male 8-week-old C57BL6J mice were purchased from CLEA Japan (Tokyo, Japan). PPARα-KO mice (strain: B6.129S4-*PparatmlGonz*/J, stock number: 008154) were purchased from the Jackson Laboratory (Bar Harbor, ME, USA). They were allowed free access to water and food, when otherwise noted.

### Plasmids for targeted DNA demethylation

Targeted DNA demethylation was performed as described previously^15^, using the pPlatTET-gRNA2 all-in-one vector, which contains dCas9-SunTag (linker length: 22 aa), scFV-sfGFP-TET1CD, and gRNA expression system. The gRNAs were selected using the CRISPR website from Dr. Feng Zhang’s laboratory (http://crispor.tefor.net). Cloning was performed using linearization of an *Afl*II site and Gibson assembly mediated incorporation of the gRNA insert fragment. The target sequences are described in Supplementary Table 1a.

### Cell culture and transfection

The Hepa1-6 cell line was purchased from the American Type Culture Collection (Manassas, VA, USA) and cultured at 37 °C in 5% CO_2_ in Dulbecco’s modified Eagle’s medium-high glucose supplemented with 10% fetal bovine serum. We transfected 15 μg of the all-in-one vector (in the case of gRNA1+2, 7.5 μg of the all-in-one vector with gRNA1 and that with gRNA2 were employed) into Hepa1-6 cells in 10-cm diameter dishes with Lipofectamine 3000 (#L3000008, Thermo Fisher Scientific, MA, USA) in accordance with the manufacturer’s protocols, harvested 48 h, and thereafter stained with 7-AAD Viability Staining (#420404, BioLegend, San Diego, CA, USA), followed by the analysis using FACSAria II (BD Biosciences, Franklin Lakes, NJ, USA) and BD FACSDiVa™ Software (BD Biosciences). We analyzed 1 × 10^4^ events using a 488 nm blue laser and 530/30 and 695/40 bandpass filters. Transfection efficiency was determined using the percentage of GFP positive cells. (Supplementary Fig. 1)^35^.

For stable transfection^36^, briefly, we linearized 15 μg of the all-in-one vector (in the case of gRNA1+2, 7.5 μg of all-in-one vector with gRNA1 and that with gRNA2 was employed) with *Apa*LI, which were cleaned using a QIAquick PCR Purification Kit (#28106 QIAGEN, Hilden, Germany) and transfected into Hepa1-6 cells in 10-cm diameter dishes with Lipofectamine 3000 (day 0). On the following day, we split the cells at 1:20 and seeded them into twenty 10-cm diameter dishes. On the third day after transfection, we added 1000 μg/ml of G418 (Geneticin) to the culture medium and replaced with fresh medium with G418 every 4–5 days. We then picked single colonies and seeded these into 24-well plates (day 21), followed by scaling up to 6-well plates. We selected cell lines by microscopic evaluation of fluorescence intensity, which were seeded into 10-cm diameter dishes, and passaged once every 3 days, followed by BS analysis (day 42).

### Bisulfite sequencing (BS) analysis

Genomic DNA was extracted from the cultured cells and mouse liver by the DNA lysis buffer (1% SDS, 0.1 M NaCl, 20 mM EDTA, 50 mM Tris-HCl pH 8.0, and Proteinase K) and an All Prep DNA/RNA Mini kit (#80204, QIAGEN), respectively. BS analysis was performed as follows: 2 µg of the genomic DNA was subjected to bisulfite treatment using an EpiTect Bisulfite Kit (#59104, QIAGEN) in accordance with the manufacturer’s instructions. Sequential PCR amplification of *Fgf21* was performed using specific primers designed with a web tool, MethPrimer (http://www.urogene.org/methprimer/)^37^, as described in Supplementary Table 2c. The reaction profiles were 40 cycles of 98 °C for 10 s, 54 °C for 30 s, and 72 °C for 120 s. The amplified fragments were ligated into pGEM-T Easy Vectors (#A1360, Promega, Madison, WI, USA), and more than 14 clones were sequenced per reaction. A web-based quantification tool for DNA methylation analysis was used for BS analysis of CpG methylation (http://quma.cdb.riken.jp/)^38^. The statistical significance of the difference between two groups of the entire set of CpG sites was evaluated using the Mann–Whitney *U*-test.

### Evaluation of off-target effects

We employed a web tool, CRISPR direct (http://crispr.dbcls.jp/), to search for off-target regions. We searched the 12 bases in the 3′-region of the target sequence adjacent to the protospacer adjacent motif using the web tool. We excluded sites unsuitable for analysis, whose sequences contained repeats or no PCR primers indicated using MethPrimer. Considering the criteria above, one and four sites for gRNA1 and gRNA2, respectively, were selected and subjected to off-target analysis as described in the previous report^16^. PCR primers are as described in Supplementary Table 2d.

### Quantitative RT-PCR analysis

Total RNA was extracted from Hepa1-6 cells using Sepasol reagent (#09379-84, Nacalai Tesque, Kyoto, Japan) and 1 μg of total RNA was used for the first-strand cDNA synthesis using ReverTra Ace (#TRT-101, Toyobo, Osaka, Japan) and Random Primer (#48190011, Thermo Fisher Scientific). The reaction profile was 30 °C for 10 min, 42 °C for 60 min, and 99 °C for 5 min. Then, 100 ng of cDNA was subjected to quantitative RT-PCR using a StepOnePlus Real-time PCR System (Applied Biosystems, Foster City, CA, USA) with Fast SYBR Green Master Mix Reagent (#4385612 Thermo Fisher Scientific). The primer sets are listed in Supplementary Table 1b. The reaction profile was 95 °C for 20 s followed by 40 cycles of 95 °C for 30 s and 60 °C for 30 s. The mRNA levels were normalized to those of 36B4 and analyzed using the comparative CT method.

For the analysis of the copy number after random integration of the dCas9 cassette, 30 ng of genomic DNA were analyzed by quantitative real-time PCR using the comparative CT method. Quantification of the dCas9 DNA was normalized to that of the single-copy nuclear gene *Ndufv1* (GenBank accession no. NM_133666) using the primer pairs, as described in previous reports^39,40^. PCR primers for *dCas9* and *Ndufv1* were as described in Supplementary Table 1c.

### siRNA transfection

For Dnmts siRNAs transfection, we purchased ON-TARGETplus Dnmt1 (L-056796-01-0005), Dnmt3a (L-065433-01-0005), Dnmt3b (L-044164-00-0005) siRNAs, and non-targeting pool (D-001810-10-20) from Dharmacon (Lafayette, CO, USA). To confirm gene expression levels of Dnmts, 20 pmol of scramble or Dnmts siRNA per well in 12-well plates were transfected into Hepa1-6 cells using Lipofectamine RNAiMAX (#13778075, Thermo Fisher Scientific) in accordance with the manufacturer’s protocols.

### Chromatin immunoprecipitation (ChIP) assay

For ChIP assays, we employed a ChIP-IT Express kit (#53008, ACTIVE MOTIF, Carlsbad, CA, USA), in accordance with the manufacturer’s protocol with some modifications. In brief, cells were transfected with 37.5 μg of the all-in-one vector using Lipofectamine 3000 (day 0), then 50 μM K-877 (a gift from Kowa Pharmaceutical Company, Nagoya, Japan) was added on day 2. On day 4, cells were fixed with 1% formaldehyde in Dulbecco’s modified Eagle’s medium-high glucose without fetal bovine serum for 10 min at room temperature on the shaker and quenched with 2.5 M glycine. Cross-linked cells were washed in phosphate-buffered saline, resuspended in lysis buffer (10% SDS, 50 mM NaCl, 10 mM EDTA, 50 mM Tris-HCl pH 8.0, and protease inhibitors) and sonicated by using a Branson 250 Digital Sonifier (#SFX250, Branson Ultrasonics Corporation, Danbury, CT, USA) at 25% power amplitude. Chromatin samples in 800-μl aliquots were incubated with Protein G-conjugated DynaBeads (#10004D, Life Technologies, Carlsbad, CA, USA) coupled with 5 μg anti-PPARα (provided by Dr. Toshiya Tanaka, Division of Metabolic Medicine, Research Center for Advanced Science and Technology, The University of Tokyo, Japan)^41^ or with appropriate antibodies (Supplementary Table 2a). We also employed rabbit (#ab171870, Abcam, Cambridge, UK) and mouse (#ab18413, Abcam) IgG as control antibodies. The dilution rate for all antibodies is described in Supplementary Table 2a. The ChIP-enriched DNA samples were analyzed by quantitative PCR using the primer sets described in Supplementary Table 2b. We calculated a percent input by dividing ChIP-enriched DNA with input DNA. Then, fold enrichment was obtained as a percent input relative to that with scramble gRNA at basal state (before K-877 administration).

### Hydrodynamic tail vein injection

We diluted 100 µg of the all-in-one vector in TransIT-EE Hydrodynamic Delivery Solution (#MIR5340, Takara Bio Inc., Shiga, Japan) to a volume equivalent to ∼10% of a mouse’s body weight (for example, 2 ml for a 20 g mouse). Eight-week-old male mice were placed in a restrainer, and their tail was placed in a 45–50 °C water bath for 40–50 s. Using a 2.5 ml syringe with a 27-gauge needle, the diluted vectors were injected within 7–8 s into the mouse tail veins as described previously^42–45^.

### Histological analysis

The livers of mice were fixed in 4% paraformaldehyde phosphate buffer solution for 24 h at 4 °C, replaced in 30% sucrose in phosphate-buffered saline for 48 h, and then embedded in optimal cutting temperature compound and frozen at −80 °C for cryosectioning. Each liver was cut into 10-μm thick sections, which were mounted on glass slides. Nuclei were stained with Hoechst 33342 (#B2261, SIGMA-ALDRICH, St. Louis, MO, USA). To evaluate the induction efficiency after HTVi, GFP-positive and Hoechst 33342-positive cells were counted with 10-fold magnification using a fluorescence microscope and Image J image-analyzing software (NIH). The induction efficiency was calculated as the number of GFP-positive cells/number of Hoechst 33342-positive cells (%).

### Biochemical assays

Serum FGF21 concentrations were determined using Rat/Mouse FGF21 enzyme-linked immunosorbent assay (ELISA) kits (#MF2100, R&D Systems, Minneapolis, MN, USA). Serum corticosterone concentrations were determined using a Corticosterone EIA Kit (#K014-H1, Arbor Assays, Ann Arbor, MI, USA).

### Statistical analysis

Data are expressed as the mean ± standard deviation (SD). Data were compared using the Mann–Whitney *U*-test. Spearman’s rank correlation coefficient was used to evaluate correlations between %DNA methylation and *Fgf21* mRNA levels. Statistical analysis was performed using Prism 7 (Graph-Pad software, Inc., La Jolla, CA, USA). Differences were considered significant at *P* < 0.05.

## Supplementary information

Supplementary information.

## Data Availability

The data that support the findings of this study are available from the corresponding authors upon reasonable request.
