# Supplementary material for: Targeted DNA demethylation of the Fgf21 promoter by CRISPR/dCas9-mediated epigenome editing
Source: Sci Rep. 2020 Mar 20;10:5181. doi: 10.1038/s41598-020-62035-6 (PMC7083849; doi:10.1038/s41598-020-62035-6)
Supplement: Supplementary file 1 — Supplementary information. [file 41598_2020_62035_MOESM1_ESM.pdf]

Supplementary information

Targeted DNA demethylation of the *Fgf21* promoter by CRISPR/dCas9-mediated epigenome editing

Nozomi Hanzawa, Koshi Hashimoto, Xunmei Yuan, Kenichi Kawahori, Kazutaka Tsujimoto, Miho Hamaguchi, Toshiya Tanaka, Yuya Nagaoka, Hiroshi Nishina, Sumiyo Morita, Izuho Hatada, Tetsuya Yamada, and Yoshihiro Ogawa

Address all correspondence to:
Koshi Hashimoto, M.D., Ph.D.
Department of Diabetes, Endocrinology and Hematology,
Dokkyo Medical University Saitama Medical Center,
2-1-50 Minami-Koshigaya, Koshigaya, Saitama 343-8555, Japan
Tel/Fax: +81-48-965-1111/8253. E-mail: k-hashi@dokkyomed.ac.jp

Supplementary Figure 1

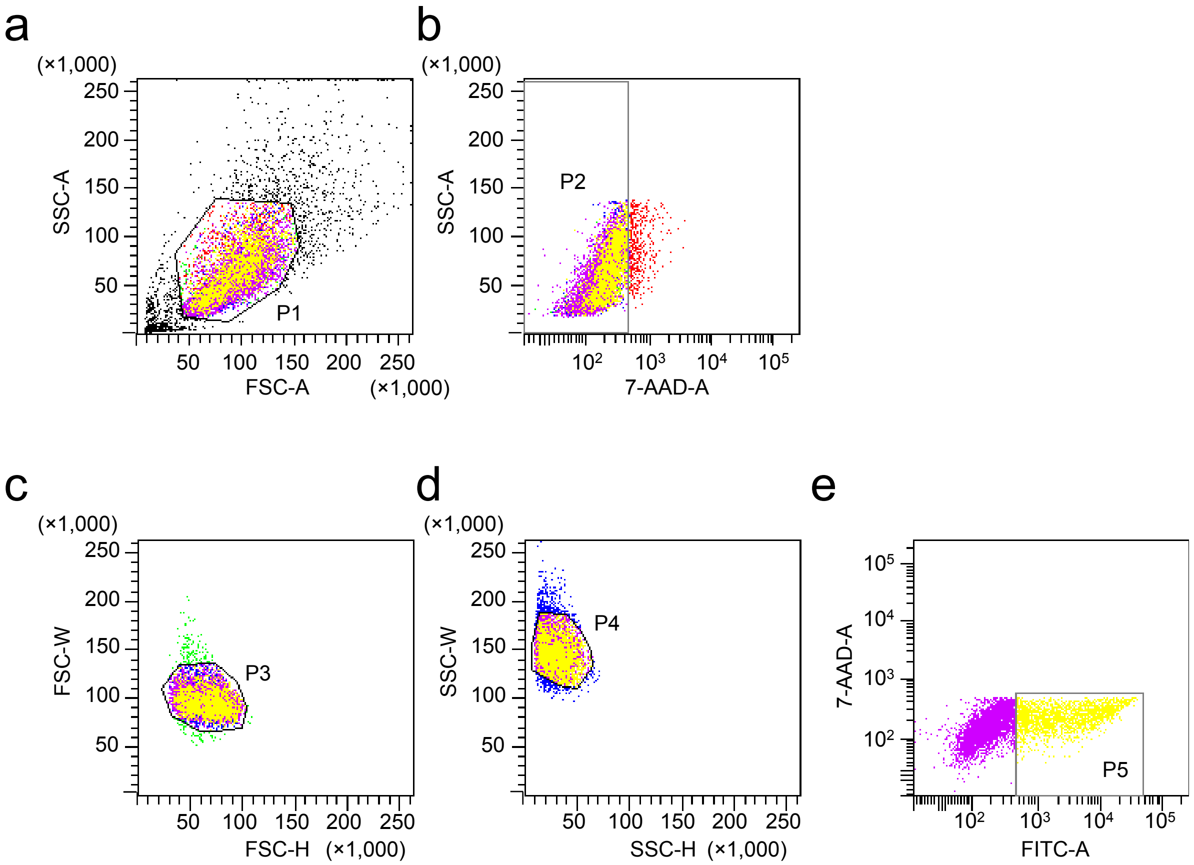

f

Population	#Events	%Parent	%Total
All Events	10,000	-	100.0
P1	8,396	84.0	84.0
P2	7,974	95.0	79.7
P3	7,782	97.6	77.8
P4	7,177	92.2	71.8
P5	1936	27.0	19.4

Supplementary Figure 2

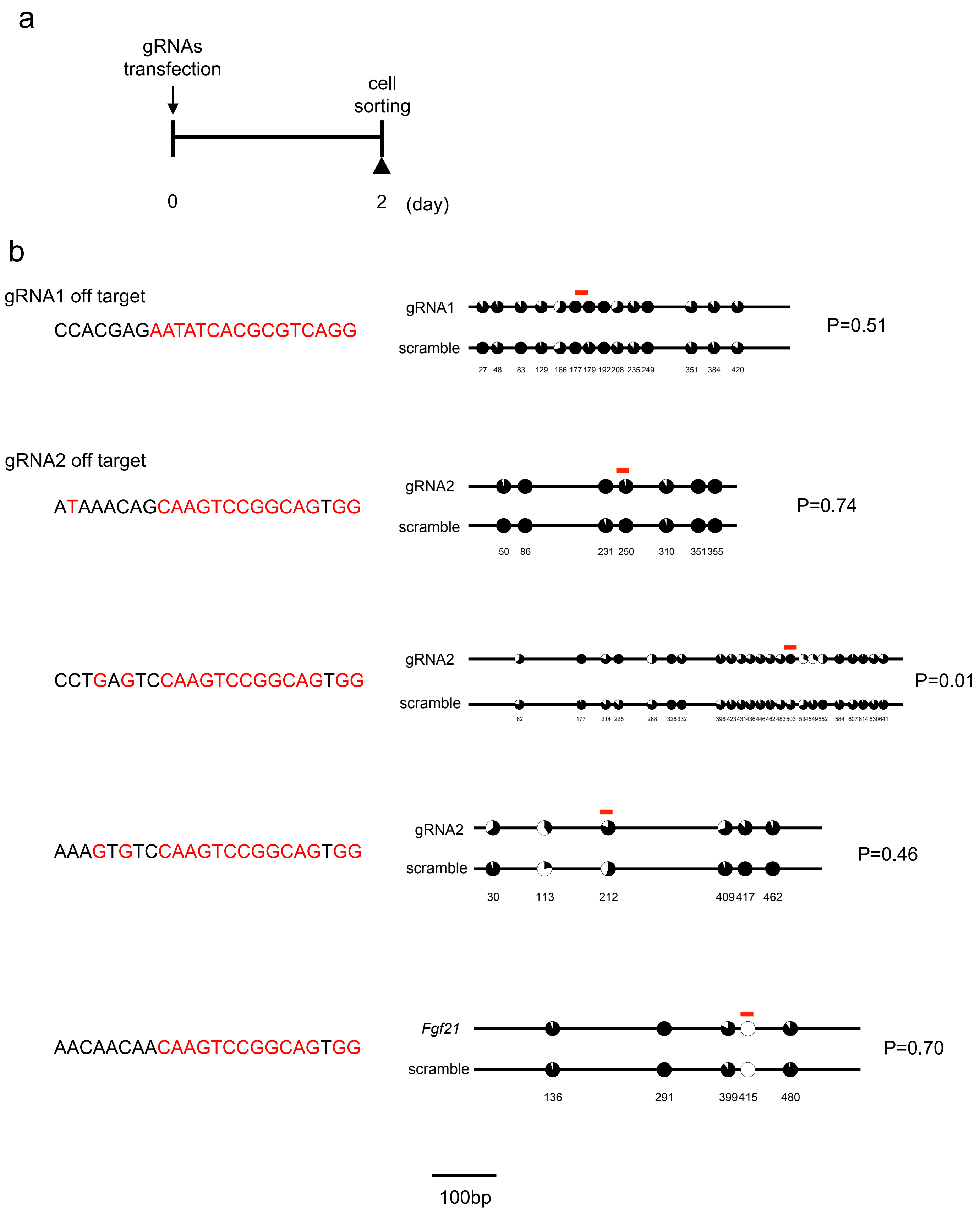

Supplementary Figure 3

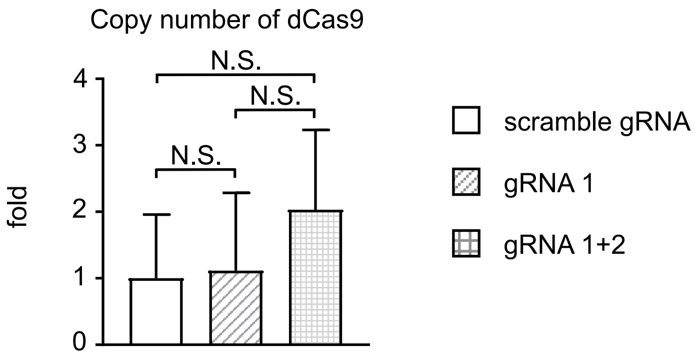

Supplementary Figure 4

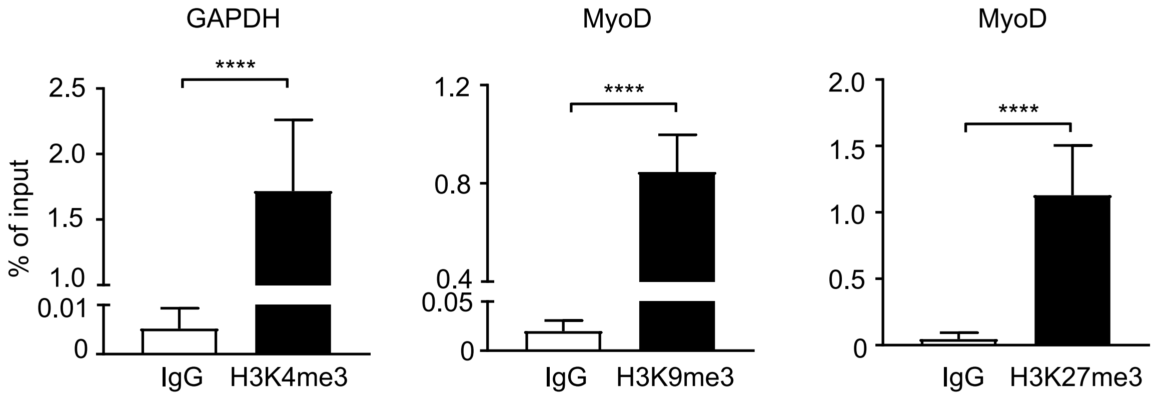

Supplementary Figure 5

a

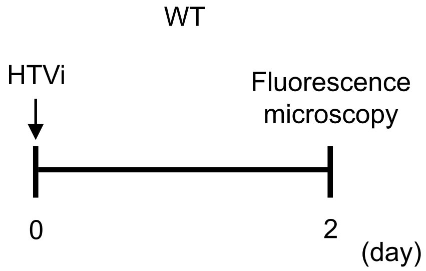

b

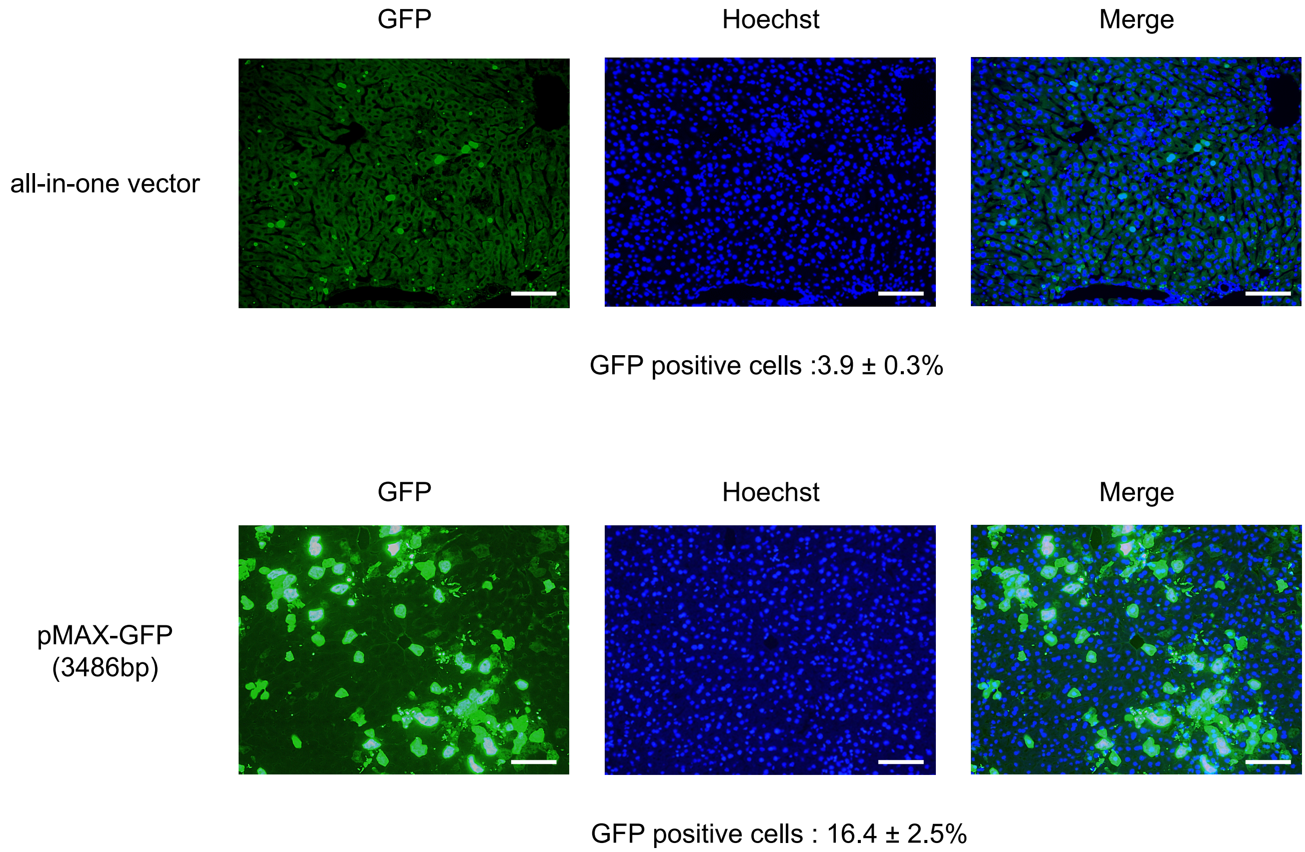

Supplementary Figure 6

a

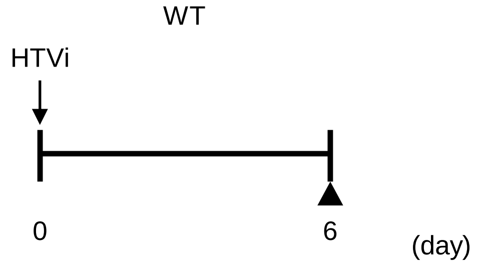

b

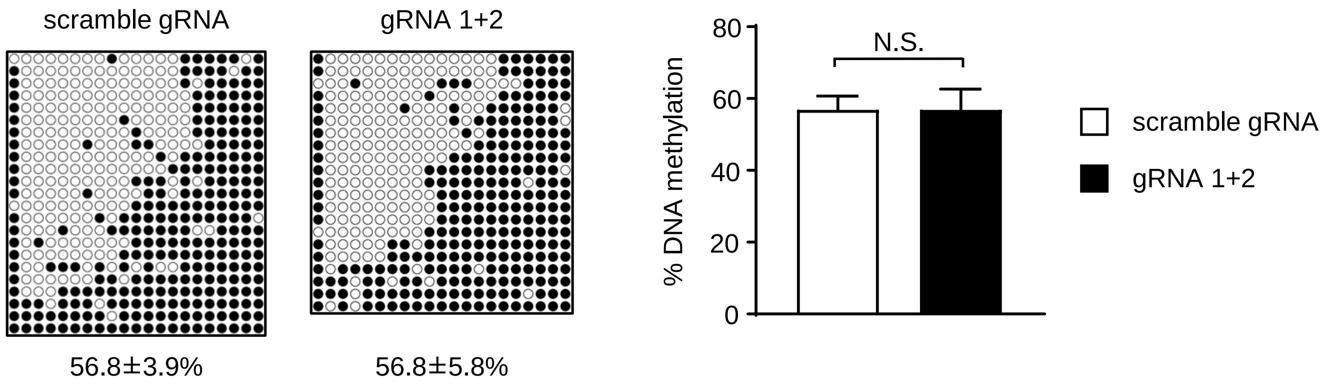

Supplementary table 1

a gRNA target sequences

Target name	Target Sequence
scramble	GCACTACCAGAGCTAACTCA
1	TGTGTCAAATATCACGCGTCAGG
2	TTGGGGGTCAAGTCCGGCAGAGG

b Primer sequences for RT-qPCR

Gene	Forward	Reverse
m36B4	GGCCCTGCACTCTCGCTTTC	TGCCAGGACGCGCTTGT
mFgf21	CCTCTAGGTTTCTTTGCCAACAG	AAGCTGCAGGCCTCAGGAT
mDnmt1	GGCTTCAGTGGCATGAAC	CTGCAGCCAAGATGATGG
mDnmt3a	CATGAACAGGCCTTTGGCA	TCTTGCAGCTCCAGCTTATC
mDnmt3b	CCAAAAGGAGGCCCATTAGAG	GTACCCCGTTGCAATTCCAT
sfGFP	TTCTTTGTTTGGTCCGGCAG	ATCCCACACCATGATGAGCA
TET1CD	GATCCGAACTGCCCACCTG	TCCTGCCCCAAGGTGTGTAT
dCas9	TGCCCCAAGTGAATATCGTG	GACTTGCCCTTTTCCACTTTG

c Primer sequences for copy number of dCas9

Gene	Forward	Reverse
dCas9	TGCCCCAAGTGAATATCGTG	GACTTGCCCTTTTCCACTTTG
Ndufv1	CTTCCCCACTGGCCTCAAG	CCAAAACCCAGTGATCCAGC

Supplementary table 2

a Antibodies used in ChIP assay

Antibody	Manufacturer	Dilution rate
anti-trimethyl-histone H3 (Lys4)	Merck Millipore, 07-473	1:267
anti-trimethyl-histone H3 (Lys27)	Abcam, ab6002	1:267
anti-trimethyl-histone H3 (Lys9)	Abcam, ab8898	1:267
anti-DNA methyltransferase (Dnmt)1	Abcam, ab13537	1:267
anti-DNA methyltransferase (Dnmt)3a	Abcam, ab2850	1:267
anti-DNA methyltransferase (Dnmt)3b	Abcam, ab2851	1:267
anti-RNA polymerase (pol) II	Abcam, ab5408	1:267
anti-rabbit IgG	Abcam, ab171870	1:300
anti-mouse IgG	Abcam, ab18413	1:300

b Primer sequences for ChIP assay

Gene	Forward	Reverse
mFgf21 (-997/-923bp)	AGGCCCGAATGCTAAGC	AGCCCAGCAGGTGGAAGTCT
mFgf21 (-106/+21bp)	TGGAATTCAGGTTCCTGCCAA	GAGAAGACACTAAGGCTGTC
Gapdh	ACCAGGGAGGGCTGCAGTCC	TCAGTTCGGAGCCCACACGC
MyoD	GCCCGCAGTAGCAAAGTAAG	GAAACCGGATCCAACTAGCA

c Primer sequences for bisulfite sequencing

Gene	Forward	Reverse
mFgf21	AGTGGTGAGAATTTAGGAGTTTTTTT	TCCTCCAAATTTAAAAATACAAACC

d Primer sequences for bisulfite sequencing (off target)

Gene	Forward	Reverse
gRNA 1 off target	TATATTTTTTTAAAGAAAGTTGTAGT	ACAACACTAATCAAATTAATATTTTT
gRNA 2 off target 1	TAAGGGAGAAAGAGGAGAAAGTTTTAT	CAAACTAACCTTAAACTCAAAAATCTACC
gRNA 2 off target 2	TTAGGAATTTTGGTTTTTTAAAGTAGGGAT	CAAATAAAACTCCATAAAAATAAAACCC
gRNA 2 off target 3	ATTTTTATTTTTTTGATGGTTAAGGAT	TTCTTTTATACTCTTTATAACCCAAACTAA
gRNA 2 off target 4	TGGGATTTGAATTTAGGATTTTTAG	CAATTCCATCACCTTTTTAATTATATTATC
